# Horizontal Gene Transfer of Pectinases from Bacteria Preceded the Diversification of Stick and Leaf Insects

**DOI:** 10.1038/srep26388

**Published:** 2016-05-23

**Authors:** Matan Shelomi, Etienne G. J. Danchin, David Heckel, Benjamin Wipfler, Sven Bradler, Xin Zhou, Yannick Pauchet

**Affiliations:** 1 Department of Entomology, Max-Planck Institute für chemische Ökologie, Hans-Knöll-Str. 8, 07745 Jena Germany; 2 INRA, Univ. Nice Sophia Antipolis, CNRS, UMR 1355-7254 Institut Sophia Agrobiotech, 06900 Sophia Antipolis, France; 3 Friedrich-Schiller-Universität Jena, Institut für Spezielle Zoologie und Evolutionsbiologie, Erbertstr. 1, 07743 Jena, Germany; 4 Johann-Friedrich-Blumenbach-Institut für Zoologie und Anthropologie, Georg-August-Universität Göttingen, Berliner Str. 28, 37073 Göttingen, Germany; 5 China National GeneBank, BGI-Shenzhen, Beishan Road, Beishan Industrial Zone, Yantian District, 518083, Shenzhen, Guangdong Province, China

## Abstract

Genes acquired by horizontal transfer are increasingly being found in animal genomes. Understanding their origin and evolution requires knowledge about the phylogenetic relationships from both source and recipient organisms. We used RNASeq data and respective assembled transcript libraries to trace the evolutionary history of polygalacturonase (pectinase) genes in stick insects (Phasmatodea). By mapping the distribution of pectinase genes on a Polyneoptera phylogeny, we identified the transfer of pectinase genes from known phasmatodean gut microbes into the genome of an early euphasmatodean ancestor that took place between 60 and 100 million years ago. This transfer preceded the rapid diversification of the suborder, enabling symbiont-free pectinase production that would increase the insects’ digestive efficiency and reduce dependence on microbes. Bacteria-to-insect gene transfer was thought to be uncommon, however the increasing availability of large-scale genomic data may change this prevailing notion.

Herbivores depend on plant cell wall degrading enzymes, such as cellulases and pectinases, to break down ingested matter. Any enzyme not produced endogenously (from the organisms’ own genomes) might be provided by symbionts residing in the gut^1^. However, occasionally horizontal gene transfer of enzymes from a microbe to an animal organism enables symbiont-independent digestion in the host^2^,^3^,^4^. While pectinase enzymes [endo- and exo-polygalacturonases] appear to rarely occur in animals, fungal and bacterial pectinase genes have been found as part of the genome of leaf beetles (Chrysomelidae) and weevils (Curculionidae), suggesting horizontal gene transfer events. These genes of putative microbial origin are expressed endogenously and have been experimentally shown to actually degrade pectin^2^,^5^,^6^.

Recently, multiple transcripts for highly expressed polygalacturonase (pectinase) genes were found in the midgut transcriptomes of six species of stick insects^7^ (Phasmatodea). The sequences showed high similarity to microbial pectinase genes, however the same genes were also found in genomic DNA from microbe-free brain tissue of these insect species^7^. This confirms that these pectinases and their eukaryote-specific signal peptides are endogenously transcribed by the insects and do not represent microbial contamination. This fact is further corroborated by their presence in multiple species reared on different diets and the fact that the phasmatodean digestive tract is unsuited for microbial fermentation^8^. Additionally, the reproductive physiology and behavior of Phasmatodea^9^ preclude vertical transmission of obligate symbionts due to the absence of egg-smearing, coprophagy, or adult-nymph trophyllaxis^10^, and both culturing and metagenomics assays have demonstrated an absence of symbiotic microbes^8^,^11^.

Therefore, it was assumed that these genes were acquired via horizontal gene transfer^7^, but definite evidence for this hypothesis was lacking and unanswered associated questions remained: What was the source of these genes? What was/is its ecological relationship to Phasmatodea? What are these enzymes’ activities? How did this gene family evolve after the transfer? Additionally, the timing of this putative horizontal gene transfer is undetermined. This is largely due to the paucity of molecular data for Phasmatodea—including the suborder Timematodea, which is the sister group of all remaining stick and leaf insects or Euphasmatodea^12^—and for closely related insect orders such as grasshoppers (Orthoptera), webspinners (Embioptera), or roaches (Blattodea)^13^.

Identifying a horizontal gene transfer requires data from the source and the recipient and/or its descendants. Additionally, outgroup taxa are needed to ensure the absence of the target gene, to determine that the horizontal gene transfer occurred within the group of interest or its common ancestor. Therefore, such findings are limited by the diversity of published sequences^14^,^15^. To address the questions outlined above, we combined biochemical analyses of these individual phasmatodean enzymes along with identification of these molecules within potential, microbial sources and potential insect hosts. To this end, we used assembled RNASeq data of phasmatodean and other polyneopteran insects sampled by the 1KITE consortium, (1K Insect Transcriptome Evolution Project, http://www.1kite.org).

## Results

### Activity of Individual Phasmatodea Pectinases

**Figure 1:**
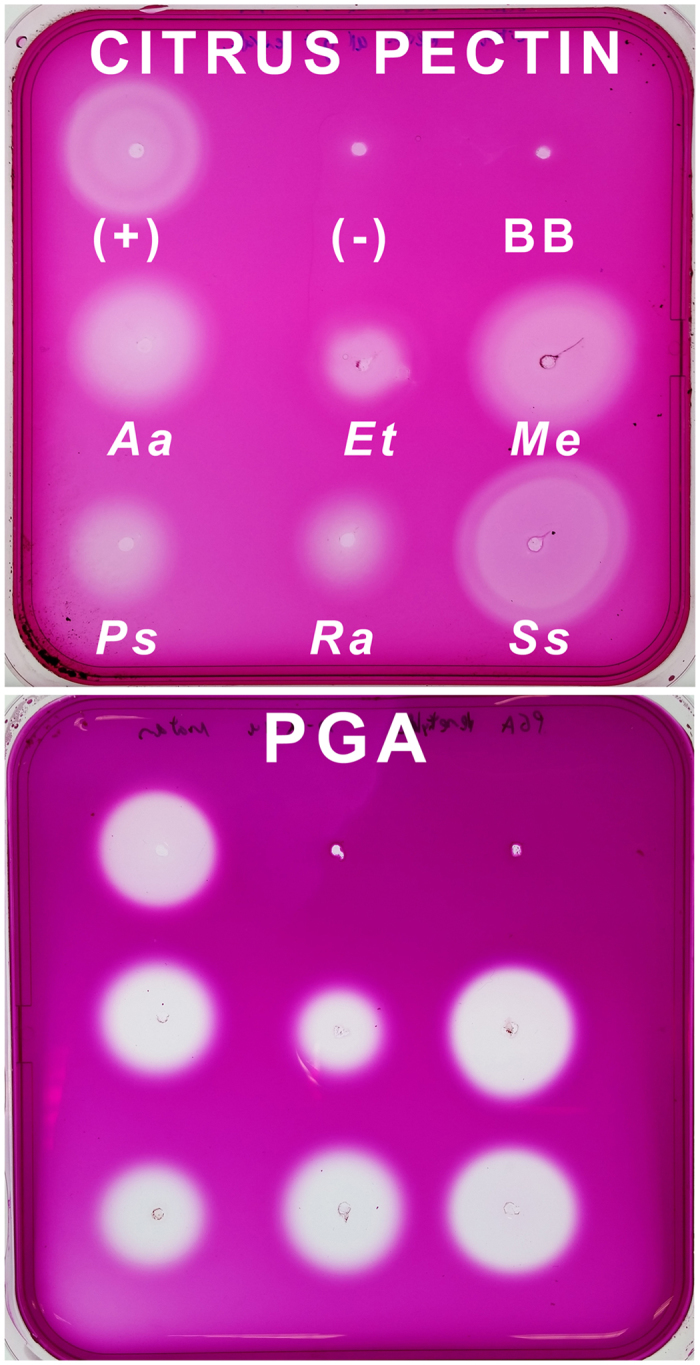
Substrate tests of stick insect gut extracts. The positive control was the gut extract of the beetle *Phaedon cochleariae* (Chrysomelidae, Coleoptera). Blackberry leaves were used to control for background enzymes in the diet, although guts were purged of contents before use in this assay. Aa = *Aretaon asperrimus*. BB = Blackberry leaves. Et = *Extatosoma tiaratum*. Me = *Medauroidea extradentata*. PGA = Polygalacturonic acid. Ps = *Peruphasma schultei*. Ra = *Ramulus artemis*. Ss = *Sipyloidea sipylus*.

**Figure 2:**
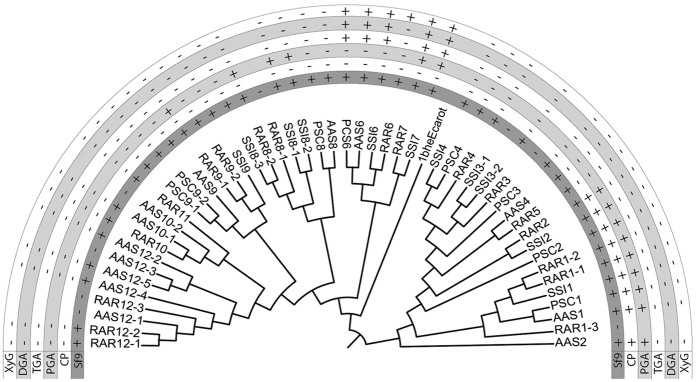
Maximum Likelihood tree and enzymatic activities of pectinases from four phasmatodean species. The outgroup is a bacteria, *Erwinia carotovora*. A “+” in the *Sf9* row means the gene was successfully amplified, transformed, and expressed into *Sf*9 cells, enabling later substrate tests. For the other rows, “+” means positive enzymatic activity and “−” means no activity detected when the expressed enzyme was tested on the given substrate. CP = citrus pectin, DGA = digalacturonic acid, PGA = polygalacturonic acid, TGA = trigalacturonic acid, XyG = xylogalacturonan.

Gut extracts from all six species with published transcriptomes^7^ could degrade citrus pectin and polygalacturonic acid (PGA) (Fig. 1). From these six species we obtained full length ORFs for 93 pectinase enzymes, including some pectinase genes the original six transcriptomes missed (Table S1). From the four species chosen for downstream expression (comprising 50 enzymes in total), we successfully amplified 44 enzymes, transformed *E. coli* with 43, and expressed 42 in *Sf*9 cells (Figure S1, Table S1). Enzyme activity as tested with thin layer chromatography (TLC) (Figures S2–S6) and agarose diffusion assays (Figures S7 and S8) mostly correlated with the gene clustering (Fig. 2): enzymes with the same activity profiles formed monophyletic clades, such that the activity of an enzyme could be predicted by its position in the tree. Several enzymes could degrade citrus pectin and/or PGA to dimers and bigger oligomers, meaning they are endo-active polygalacturonases. Most enzymes in one monophyletic group could degrade PGA and the oligomers into galacturonic acid. This implies that they are exo-active enzymes. Enzymes in this clade were also active on xylogalacturonan. Other enzymes showed no detectable activity on any substrate, including enzymes known to be highly expressed such as PSC3, which is the most highly expressed pectinase and 10^th^ most highly expressed enzyme in the *Peruphasma schultei* anterior midgut^7^.

### Evidence for Horizontal Gene Transfer

**Figure 3:**
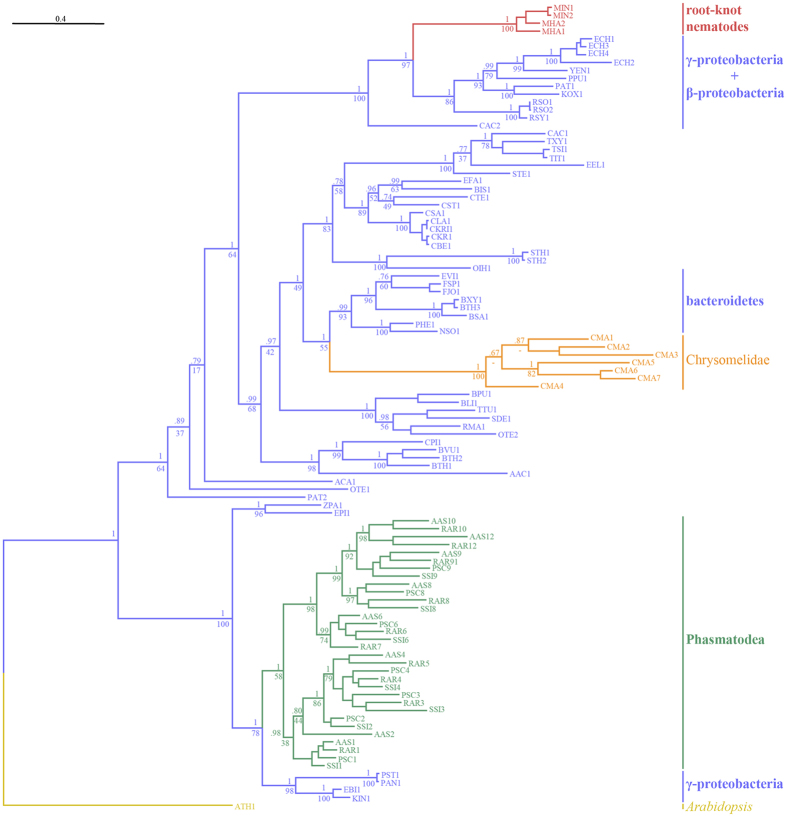
Phylogeny of the GH28 proteins. To minimize the size of the tree, only pectinases from the four exemplar species of Phasmatodea used in the expression assays are included in this figure, with one representative from genes with multiple alleles. Gene tree topology obtained from the Bayesian analysis, (posterior probability support at nodes) chosen as a reference topology. Bootstrap values obtained from the ML analysis are indicated at the corresponding nodes below posterior probability values. The tree is represented as a cladogram with branch lengths and rooted with the *A. thaliana* sequence (GenBank Accession Number NP_850359.1) as outgroup. Branches are colored in violet if derived from bacterial sequences, in red if derived from nematode sequences, in orange if derived from beetle sequences, in green if derived from Phasmatodea sequences, and in yellow for the plant sequence outgroup. Species name abbreviations are indicated in Table S2.

The Bayesian analysis for insect, nematode, and microbial GH28 pectinases converged after 300,000 generations with an average standard deviation of split frequencies of 0.014. The alpha-shape parameter of the gamma distribution was 1.8. Due to the bootstrap algorithm in RAxML, the Maximum Likelihood (ML) analysis converged after 450 bootstrap replicates. The Le and Gascuel model (LG) was determined as best-scoring evolutionary model; the estimated alpha-shape parameter of the gamma distribution was 1.7. The ML and Bayesian phylogenetic analyses converged, providing trees with almost the same topology. The Bayesian topology was chosen as reference topology, and the more conservative^16^,^17^ bootstrap support values obtained from the ML analysis were indicated on the corresponding branches (Fig. 3).

This consensus pectinase phylogeny shows that genes of the GH28 family pectinases originated independently three times within the studied Metazoa. Root-knot nematode pectinases^18^ (*Meloidogyne* sp.) form a highly supported, monophyletic clade (posterior probability [PP]: 1.0, bootstrap support [BS]: 100) and are nested within gamma-proteobacteria, beta-proteobacteria, and Firmicutes (PP: 1.0, BS: 97). Pectinase genes identified in the leaf beetle *Callosobruchus maculatus*^5^ (Chrysomelidae, Coleoptera) form a monophyletic clade with maximal PP and BS support that clusters with Bacteroidetes (PP: 1.0, BS: 55). Finally, the phasmatodean pectinase genes form a third, distinct and well-supported clade (PP: 1.0, BS: 58), with two monophyletic sub-clades within. Their closest related sequences are a group of gamma-proteobacteria, including the genera *Pantoea, Klebsiella*, and *Enterobacter* (PP: 1.0, BS: 78).

### Pectinases in the Polyneoptera

**Figure 4:**
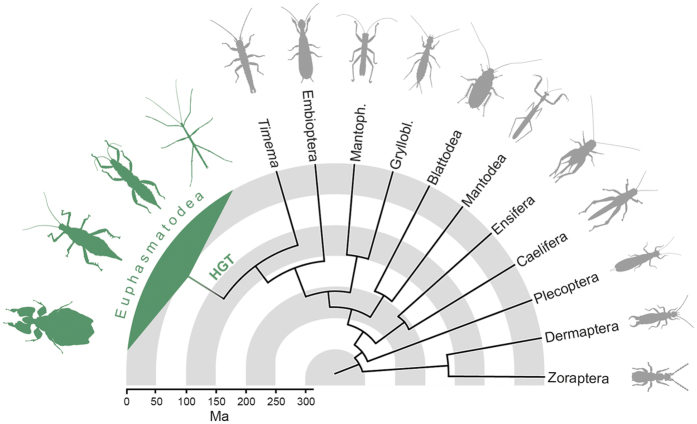
Presence of endogenous pectinases in polyneopteran species. The horizontal gene transfer (HGT) occurred after Euphasmatodea and Timematodea [identified by the single representative genus, *Timema*] diverged. Polyneopteran phylogenetic relationships and divergence times (in millions of years ago) are based on Misof *et al*.^20^ and Bradler *et al*.^12^.

From the 1KITE transcriptome assemblies, we found pectinase transcripts in all phasmatodean insects in which relevant parts of the midgut were present, except *Timema cristinae* (Fig. 4, Table S3). To verify this finding, we also mined the draft genome for this species^19^, and found it also lacks pectinase genes. This confirms that pectinase genes are indeed probably absent in *Timema*. In addition, we could not identify any pectinases in the transcriptome assemblies of any other polyneopteran insects analyzed (Fig. 4, Table S3), implying that pectinases are either not expressed or were missing due to a very low expression level of respective genes, though the latter is unlikely for digestive enzymes at the sequence depth (~2.5 Gbases) used to generate these transcriptomes^20^.

## Discussion

Although most herbivorous insects depend on symbionts in their digestive system to supply the required cellulase and/or pectinases enzymes to fully digest the plant cell walls, many do produce a subset of the necessary enzymes endogenously. It was long assumed that any enzymes not provided by symbionts are ancestral in insects^21^. This had been confirmed for GH9 cellulase, which is endogenously produced in various insect lineages^21^ including Phasmatodea^7^,^22^ and has even been hypothesized to be ancestral to all Metazoa^23^. In contrast, endogenous pectinase genes are only found in a few, distantly related insect lineages such as several groups of beetles^5^,^24^,^25^, leafhoppers^26^, aphids^27^, and phasmids^7^. In the present study, we identified, isolated, and successfully expressed multiple, endogenous, phasmatodean pectinase genes. They are found in representatives of all major phasmatodean lineages with the exception of Timematodea, which is the sister group to Euphasmatodea^12^,^19^. The pectinase genes are also absent in closely related groups such as grasshoppers, termites, and roaches (Fig. 4, Table S3; for a detailed discussion of the phylogeny of this lineage, see Beutel *et al*.^13^). The presence of endogenous pectinase is thus an evolutionary novelty for Euphasmatodea (i.e. all phasmids except Timematodea). Based on this finding and the fact that the most similar homologues to the phasmatodean pectinase genes are found in gamma-proteobacteria (Fig. 3), we conclude that the phasmatodean genes have been acquired from bacteria by horizontal gene transfer. A similar origin was assumed in beetles^5^ and nematodes^15^. However, our analysis indicates that phasmatodean pectinase genes are not closely related to those found in beetles or nematodes (Fig. 3). This implies several independent horizontal transfers for these genes in insects and in Ecdysozoa. Gamma-proteobacteria are the predominant microbes in the phasmatodean digestive tract^8^,^11^, which could suggest a symbiotic relationship, except that representatives of this group such as *Enterobacter* were also isolated from the rearing environment of Phasmatodea^8^ and were most likely transients obtained with the food. We therefore cannot clarify whether the genes identified in Euphasmatodea had been acquired from true symbionts or from microbes that occasionally occurred in the ancestral euphasmatodean digestive system. The alternative scenario, that all ecdysozoan or insect pectinase genes were inherited from a common ancestor and have been lost or are unexpressed in all other clades, is considerably less parsimonious. Furthermore, this alternative hypothesis would imply monophyly of ecdysozoan pectinases, which is not the case as they form at least three distinct clusters interspersed by bacterial homologs (Fig. 3).

The phylogeny of the euphasmatodean pectinase genes (Figs 2 and 3) suggests that originally only one or two genes or gene copies were transferred from bacteria. Prior to a later diversification of Euphasmatodea, these genes underwent further duplication and subfunctionalization^28^. Some copies retained the ancestral endo- and/or exo-acting activities, respectively capable of breaking down the polymers or smaller oligomers of galacturonic acid. Other gene copies most likely lost the ancestral pectinolytic functions, since we did not observe any activity on the substrates tested. Nevertheless, the high expression levels for these enzymes^7^ suggests they still have a (yet unknown) function. All active enzymes examined retained their canonical active sites, which are common to polygalacturonases^29^. Moreover, some inactive enzymes also retained active sites, so their inability to degrade the substrates provided remains to be explored (Table S1). For example, surrounding amino acid substitutions may modify the structure of the protein and prevent the active sites from being accessible to the substrate.

Phasmid pectinase enzyme activities were susceptible to the degree of methylation of the polygalacturonic acid substrate, raising the question of whether or not some need a pectin methylesterase enzyme (PME) to efficiently break down pectin. Bacterial pectinases in particular cannot degrade highly methylated pectins and depend on synergistic PME activity^30^, so finding these patterns in horizontally acquired pectinases in Phasmatodea is logical. However, a search for transcripts homologous to known PMEs (GenBank Accession No’s: AAM39440.1, ADO57389.1, AAK81304.1, AEE33008.1) was negative for all phasmatodean lineages. The same was observed for nematodes^15^. The possibility remains in Phasmatodea that other genes, perhaps even some of the otherwise inactive pectinases, have developed PME activity as a neofunctionalization. Alternatively, either symbiotic microbes in the phasmatodean gut are producing PMEs or the ingested plants’ own PMEs are still active in the stick insect gut. These hypotheses are presently being tested.

Timematodea contain only a single genus, *Timema*, with 21 described species found in the western United States^31^. Euphasmatodea split from Timematodea, which lack pectinase genes, between 125 (±60) and 103 (±19) million years ago^12^,^20^. The more than 3,000 described euphasmatodean species started to rapidly diversify from a common ancestor around 61 (±14) million years ago^12^,^32^. Thus, the horizontal gene transfer most likely took place within this range of ~40 million years between the split of Phasmatodea and the diversification of Euphasmatodea (Fig. 4). It remains an intriguing question whether the acquisition of endogenous pectinase genes may have influenced or even enabled this strong and rapid diversification of Euphasmatodea. This hypothesis was also proposed by Kirsch *et al*.^5^, who assumed that the acquisition of pectinase genes was a key event in the evolution of various herbivorous beetle groups. The alternative hypothesis—that the pectinase transfer occurred in an ancestor of all Phasmatodea and was subsequently lost in Timematodea—is less parsimonious but otherwise cannot be ruled out.

It is noteworthy that all insect clades for which pectinases have been identified are associated with a strictly plant-based diet. In sucking insects such as aphids^27^ and leafhoppers^26^, pectinases enzymes are involved in plant penetration^33^ and softening of the plant before oviposition^34^. Chewing insects like beetles and most likely phasmids use the enzymes for digestion of ingested plant matter^21^. Further studies may reveal whether such evolutionary events as horizontal gene transfer were present for other organisms that evolved similar dietary specialization.

The horizontal gene transfer from a transient microbe into its commensal host, as suggested here, demonstrates the various, unpredictable, and exciting pathways evolution can take. Our results show that pectinase genes were transferred from microbes to ecdysozoans several times independently, indicating that these cross-domain horizontal gene transfers may be much more common than previously thought^15^,^35^. If this can be verified in future research, the historical and present role of symbionts or transients in driving evolutionary diversification in their hosts must be reconsidered.

## Materials and Methods

### Whole midgut agarose diffusion assay for substrate activity

To test if Phasmatodea guts were pectinolytic, we filled square Petri-dishes with 0.1% solutions of either citrus pectin (Sigma) or polygalacturonic acid (PGA) (Megazyme) in 0.4% agarose and 50 mM citrate-phosphate buffer (pH 5.0). We made wells in the plates and filled them with 5 μL of macerated, whole midguts cleared of their contents and dissected from the aforementioned six species with the published midgut transcriptomes^7^: *Aretaon asperrimus* (Heteropteryginae), *Peruphasma schultei* (Pseudophasmatinae), *Sipyloidea sipylus* (Necrosciinae), and *Extatosoma tiaratum* (Lanceocercata: Extatosomatinae), *Medauroidea extradentata*, and *Ramulus artemis* (Clitumninae). We used pectinases from *Aspergillus niger* (Sigma) as positive control. Plates were incubated upside-down at 40 °C overnight, stained for one hour in 0.01% Ruthenium Red (Colour Index No. 77800) on a shaker at 20 rpm, and destained in diH2O. Enzyme activity was detectable as clearings in the stained gel.

### Creating cDNA libraries and cloning of full length genes

Amino acid sequences from known pectinases of the glycoside hydrolase (GH) family 28 (www.cazy.org) were retrieved from GenBank (Accession Numbers: JQ728556.1, Y17906.1, EU450666.1). We used the tBLASTn algorithm^36^ with an e-value cutoff of 1E-10 to mine for homologous sequences to these from the six published phasmatodean midgut transcriptomes (Genbank Accession No. PRJNA238833 & PRJNA221630). For incomplete transcripts, we designed specific primers for 5′- and 3′-Rapid Amplification of cDNA Ends (RACE) PCR using the Primer3 program v0.4.0 (http://bioinfo.ut.ee/primer3-0.4.0/primer3/). From living, cultured specimens of these six species, we dissected the anterior midgut, removed the gut contents, and stored the tissue in RNAlater^®^ solution (Qiagen). After maceration in a frozen Tissue Lyser, RNA was extracted using the innuPREP RNA MiniKit (Analytik-Jena) and purified with theRNeasy^®^ MinElute^®^ cleaning kit (Qiagen) following the manufacturers’ protocols. From the RNA, we synthesized cDNA and performed RACE PCR as needed with the SMARTer RACE cDNA Amplification Kit (BD Contech) following the manufacturer’s instructions. PCR products were cloned into One Shot^®^ Top10 Chemically Competent *E. coli* cells with the pCR™4-TOPO/TA^®^ Vector (Invitrogen), and subsequently sequenced by the Sanger method using M13 forward and reverse primers on an ABI 3730 xl automatic DNA sequencer (PE Applied Biosystems). Once we obtained complete open reading frames (ORFs) for every pectinase gene, they were converted to amino acid sequences and checked for eukaryote-specific signal peptides using the SignalP 4.1 server (http://www.cbs.dtu.dk/services/SignalP/). We annotated the sequences accordingly and deposited them in GenBank (Accession Numbers in Table S1).

### Multiple alignment and inference of pectinase gene tree

For a general pectinase tree, the phasmid proteins were combined with those of bacteria, nematodes, and beetles as identified using an NCBI database search for glycoside hydrolase (GH) family 28 enzymes, which have a conserved, GH28 pectinolytic domain (Table S2), with *Arabidopsis thaliana* (GenBank Accession Number NP_850359.1) as outgroup^37^. These GH28 sequences were aligned using MAFFT^38^ with the L-INS-i option optimized for alignment of protein sequences with one conserved domain and allowing for long gaps whenever necessary. The alignment was pruned using trimAL^39^ to remove all positions containing more than 70% of gaps. The pruned alignment was used to infer gene trees with both, Bayesian and Maximum Likelihood (ML) methods. For the Bayesian tree we used MrBayes^40^ 3.2.6 with an estimated gamma distribution among site rate variation (ASRV), along with a mixture of evolutionary models. A total of 300,000 generations were performed on 8 Monte Carlo Markov Chains (MCMC). We discarded a burn-in of 25% of sampled generations for inference of a consensus tree and calculation of posterior probabilities. The ML phylogeny was obtained using RAxML^41^ 8.1.24 using the automated selection (AUTO) of the best fitting evolutionary model and an estimated gamma distribution of ASRV. The autoMRE option (default 0.03) was used to automatically conduct bootstopping, indicating that enough bootstrap replicates had been sampled to obtain bootstrap value convergence among the tree topologies. In both cases, the *A. thaliana* GH28 sequence^37^ was used as outgroup to root the trees. The trees were visualized and exported as figures using FigTree software (http://tree.bio.ed.ac.uk/software/figtree/).

### Expression of pectinase genes in specific insect tissue

We chose four exemplar species, one from each taxonomic family, for downstream analysis: *A. asperrimus, P. schultei, R. artemis*, and *S. sipylus*. We designed gene-specific forward and reverse primers (Table S1) to amplify the complete ORF of each putative enzyme from the cDNA, and cloned them into Top10 cells with the pIB/V5-His TOPO/TA^®^ vector (Invitrogen). We included Kozak sequences (RCCATGG) at the 3′ end of the forward primers and did not include the stop codon in the reverse primers. Colony PCR or direct sequencing was done to ensure the genes were cloned in the correct direction, then we extracted the plasmids with a GeneJET™ Plasmid Miniprep Kit (Thermo Scientific) and transfected them into Sf9 cells (Invitrogen) using the reagent FuGENE HD (Promega). Culture medium was harvested after 72 hours incubation at 27 °C and centrifuged, and the supernatant tested for successful expression via Western Blot (Figure S1) with anti-V5-HRP antibody (Invitrogen). Plate assays for substrate activity were performed on the individual enzymes following the same protocol as the whole gut extracts.

### Thin Layer Chromatography (TLC) assays for substrate activity

Enzyme solutions were dialyzed in three baths of 50 mM citrate-phosphate buffer pH 5.0 at 4 °C using Slide-A-Lyzer Dialysis Cassettes (Thermo Scientific) with 10 KDa cutoffs, desalted in Zeba_TM_ Desalt Spin Columns (Thermo Scientific) with 7 KDa cutoffs, and stored at 4 °C until use. 10 μL of desalted enzyme were combined in microcentrifuge tubes with 2 μL 0.2 M citrate phosphate buffer (pH 5.0) and 8 μL of the following ratios of 1% w/v substrate stock solutions and diH2O: 4:4 citrus pectin (Sigma), 4:4 soy- or potato- rhamnogalacturonan (Megazyme), 4:4 demethylated polygalacturonic acid (PGA) from citrus (Sigma), 1:7 trigalacturonic acid (TGA) (Megazyme), 2:6 digalacturonic acid (DGA) (Megazyme), and 4:4 xylogalacturonan produced following the protocol published by Beldman *et al*.^25^. We used pectinases from *Aspergillus niger* (Sigma) as positive control. The tubes were incubated for 16 hours at 40 °C, then spotted onto TLC plates (silica gel 60, 20 × 10 cm, Merck) and developed with 9:3:1:4 of ethyl acetate:acetic acid:formic acid:water. We used as reference standards 2 μg each of galacturonic acid, DGA, and TGA, as well as xylose mono-, di-, and trimers and galactose as needed (Megazyme). The dried plates were sprayed with 0.2% (w/v) orcinol in 9:1 methanol/sulfuric acid, and subsequently heated with a heat gun until spots appeared.

### Timing the origin of the phasmatodean pectinases

As we did for the six transcriptomes, we mined the transcriptomes of 38 phasmatodean species, which broadly represent all recognized major lineages^32^, and 16 representative polyneopteran outgroups (GenBank Accession No: PRJNA183205, Table S3) for target genes. Digestive enzymes in stick insects are expressed in the anterior midgut^7^, which starts approximately at the thorax/abdomen border^8^. For most of the studied phasmids, the transcriptome was not taken from the entire animal but rather from the head and parts of the thorax. Table S3 provides a detailed list of which body parts were used for respective species. To ensure that the relevant midgut tissue had been included, we also mined for endogenous insect cellulase enzymes^23^ from GH family 9, which are also highly and differentially expressed in the anterior midgut^7^,^22^. Phasmids should have more than five genes from this group^7^. We therefore excluded in the present study all phasmatodean transcriptomes with five or fewer GH9 cellulase genes, which suggested the transcriptome did not include midgut tissue. For all outgroup taxa, the entire animal was used to generate the transcriptomes, ensuring the presence of all digestive tissue. To date the origin of the horizontal gene transfer we mapped the presence of endogenous pectinase genes on the dated phylogenies of Misof *et al*.^20^ and Bradler *et al*.^12^.

### Ethics

All methods were carried out in accordance with local guidelines for animal research. All experimental protocols were approved by the Max Planck Institute for Chemical Ecology in accordance with these guidelines.

### Data accessibility

Phasmatodea pectinase sequences are available under GenBank Accession Number’s KT921897-KT921989. 1KITE RNASeq data (BioProject PRJNA183205) is available from the Consortium upon request, with the Accession Numbers for published individual transcriptomes in Table S3.

## Additional Information

**How to cite this article**: Shelomi, M. *et al*. Horizontal Gene Transfer of Pectinases from Bacteria Preceded the Diversification of Stick and Leaf Insects. *Sci. Rep.* **6**, 26388; doi: 10.1038/srep26388 (2016).

## Supplementary Material

Supplementary Information

Supplementary Information
