# Author Correction: Horizontal Gene Transfer of Pectinases from Bacteria Preceded the Diversification of Stick and Leaf Insects

**DOI:** 10.1038/s41598-021-94743-y

**Published:** 2021-07-26

**Authors:** Matan Shelomi, Etienne G. J. Danchin, David Heckel, Benjamin Wipfler, Sven Bradler, Xin Zhou, Yannick Pauchet

**Affiliations:** 1 Department of Entomology, Max-Planck Institute für chemische Ökologie, Hans-Knöll-Str. 8, 07745 Jena, Germany; 2 INRA, Univ. Nice Sophia Antipolis, CNRS, UMR 1355-7254 Institut Sophia Agrobiotech, 06900 Sophia Antipolis, France; 3 Friedrich-Schiller-Universität Jena, Institut für Spezielle Zoologie und Evolutionsbiologie, Erbertstr. 1, 07743 Jena, Germany; 4 Johann-Friedrich-Blumenbach-Institut für Zoologie und Anthropologie, Georg-August-Universität Göttingen, Berliner Str. 28, 37073 Göttingen, Germany; 5 China National GeneBank, BGI-Shenzhen, Beishan Road, Beishan Industrial Zone, Yantian District, Shenzhen, 518083 Guangdong Province China

Correction to: *Scientific Reports* 10.1038/srep26388, published online 23 May 2016

This Article contains an error in the Materials and Methods section, subheading “Expression of pectinase genes in specific insect tissue” where,

“We included Kozak sequences (RCCATGG) at the 3′ end of the forward primers and did not include the stop codon in the reverse primers.”

should read:

“We included Kozak sequences (RCCATGG) at the 5′ end of the forward primers and did not include the stop codon in the reverse primers.”

