# Supplementary material for: Horizontal Gene Transfer of Pectinases from Bacteria Preceded the Diversification of Stick and Leaf Insects
Source: Sci Rep. 2016 May 23;6:26388. doi: 10.1038/srep26388 (PMC4876471; doi:10.1038/srep26388)

Supplementary Data

Horizontal Gene Transfer of Pectinases from Bacteria Preceded the Diversification of Stick and Leaf Insects

Matan Shelomi[1], Etienne G.J. Danchin[2], David Heckel[1], Benjamin Wipfler[3]*, Sven Bradler[4], Xin Zhou[5]*, Yannick Pauchet[1]

1. Department of Entomology, Max-Planck Institute für chemische Ökologie, Hans-Knöll-Str. 8, 07745 Jena Germany
2. INRA, Univ. Nice Sophia Antipolis, CNRS, UMR 1355-7254 Institut Sophia Agrobiotech, 06900 Sophia Antipolis, France.
3. Friedrich-Schiller-Universität Jena, Institut für Spezielle Zoologie und Evolutionsbiologie, Erbertstr. 1, 07743 Jena, Germany
4. Johann-Friedrich-Blumenbach-Institut für Zoologie und Anthropologie, Georg-August-Universität Göttingen, Berliner Str. 28, 37073 Göttingen, Germany
5. China National GeneBank, BGI-Shenzhen, Beishan Road, Beishan Industrial Zone, Yantian District, Shenzhen, Guangdong Province, China 518083

*members of the 1KITE consortium (www.1kite.org)

Corresponding Author: Matan Shelomi, Department of Entomology, Max-Planck Institute für chemische Ökologie, Hans-Knöll-Str. 8, 07745 Jena Germany, EMAIL: mshelomi@ice.mpg.de, TEL: +49 01749614258

Supplementary Data Legends

Figure S1: Western blots confirming the successful expression of phasmid pectinases into *Sf*9 cells. Marker (MW) used was PageRuler™ Plus Prestained Protein Ladder.

Figure S2: TLC plates for individual enzyme activity on citrus pectin. 10μl of desalted enzyme, water (negative control, -), or pectinase from *Aspergillus niger* (positive control, +) were incubated for 16 hours in microcentrifuge tubes with 1% w/v substrate in water, then spotted onto silica gel plates. Gels were stained with 0.2% (w/v) orcinol in 9:1 methanol/sulfuric acid and developed with a heat gun. Bands indicate galacturonic acid tri-, di-, or mono-mers corresponding to the markers on the left of each row, with monomers at the top and larger oligomers on the bottom. Numbers represent each enzyme as in Table S2. AAS=*Aretaon asp*errimus, ga=galacturonic acid #-mers, PSC=*Peruphasma schultei*, RAR=*Ramulus artemis*, SSI=*Sipyloidea sipylus*.

Figure S3: TLC plates for individual enzyme activity on polygalacturonic acid. Same as Figure S2.

Figure S4: TLC plates for individual enzyme activity on trigalacturonic acid. Same as Figure S2.

Figure S5: TLC plates for individual enzyme activity on digalacturonic acid. Same as Figure S2.

Figure S6: TLC plates for individual enzyme activity on xylogalacturonan. Same as Figure S2, except bands can indicate galacturonic acid, galactose, or xylose tri-, di-, or mono-mers corresponding to the markers on the right. ga=galacturonic acid #-mers, gal=galactose, xy=xylose #-mers

Figure S7: Agarose diffusion assays of the activity of individual pectinase enzymes against citrus pectin. Enzymes were incubated overnight in wells made in gels of 0.1% substrate in 0.4% agarose (pH 5.0). Plates were stained with ruthenium red and enzymatic activity was detectable as clearings in the stained gel. We used pectinases from *Aspergillus niger* (Sigma) as positive control (+) and MilliQ water as negative control (-). Multiple wells with the same ID represent different *Sf*9 cell batches, as multiple transfection attempts were performed and tested together. Numbers correspond to the enzyme IDs in Table S1.

Figure S8: Agarose diffusion assays of the activity of individual pectinase enzymes against polygalacturonic acid. Same as Figure S7.

Table S1: Pectinase genes from six phasmatodean species and the activities of those from four exemplar species. If a new gene was identified from the transcriptome, its original sequence ID is given in brackets. Only if gene isolation was successful, primers are listed. The amino-acid sequences are from four conserved regions of the *E. carotovora* pectinase, with the original sequences being NTD, GDD, GHD, and RIK. The reads per kilobase of transcript per million mapped reads (RPKM) for the *P. schultei* enzymes are provided, with their rank for most highly expressed midgut transcript[7]. CP=citrus pectin, DGA=digalacturonic acid, PGA=polygalacturonic acid, TGA=trigalacturonic acid, XyG=xylogalacturonan.

Table S2: Pectinase genes from the non-Phasmatodea used in Figure 4. Accession Numbers provided.

Table S3: Polyneoptera transcriptomes from the 1KITE project. Taxonomy is according to the Phasmida Species File (phasmida.speciesfile.org/) with amendments from Bradler et al.[12,32]. "Lib-Identified": 1KITE library identification number. Listed is the tissue used to generate the transcript libraries (whole animal for *Timema* and non-phasmatodean Polyneoptera, partial for the remaining species). Numbers of GH28 (pectinase) and GH9 (cellulase) transcripts were identified using tBlastn.

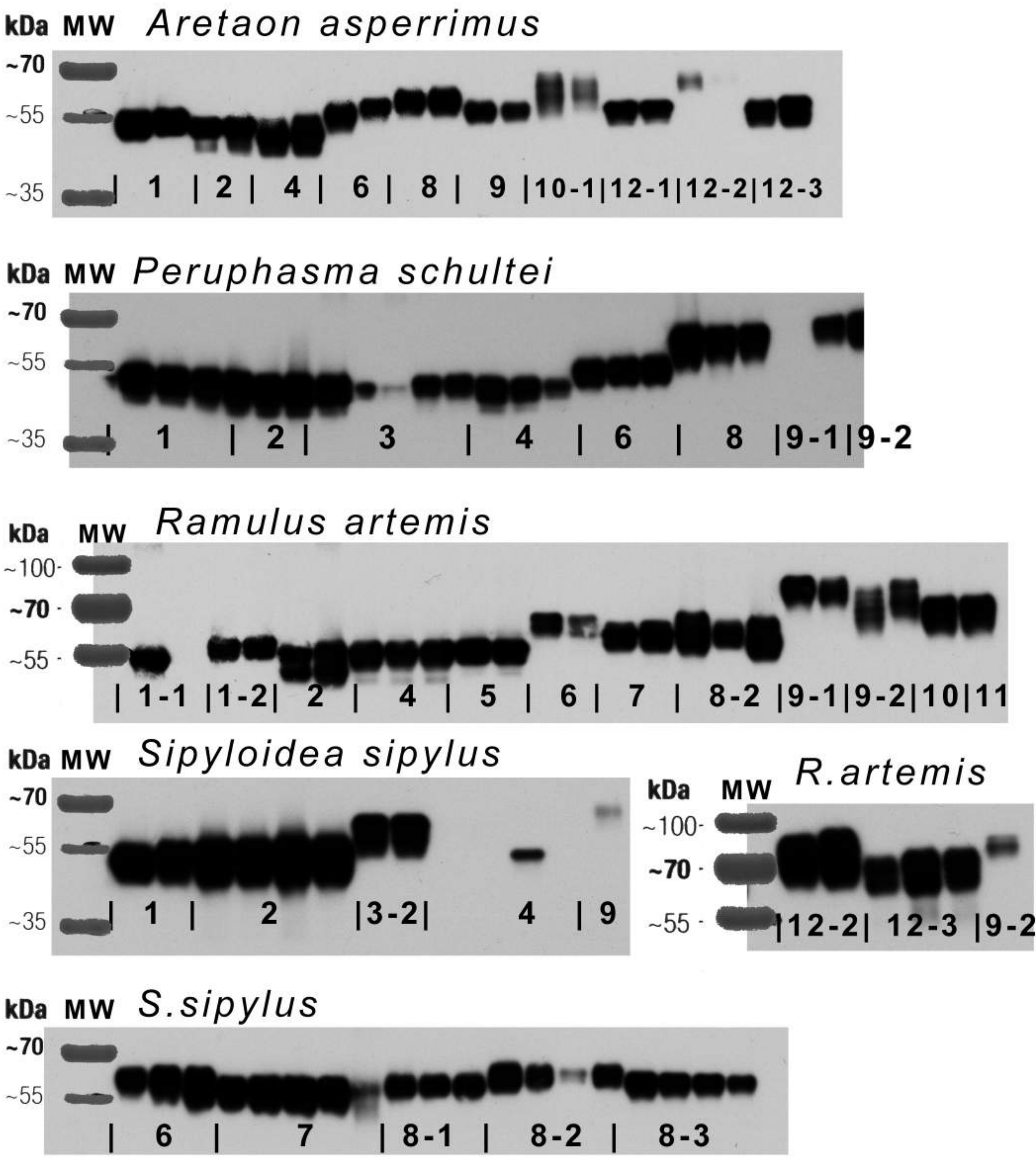
kDa MW Aretaon asperrimus
~70
~55
~35
1 2 4 6 8 9 10-1 12-1 12-2 12-3
kDa MW Peruphasma schultei
~70
~55
~35
1 2 3 4 6 8 9-1 9-2
kDa MW Ramulus artemis
~100
~70
~55
1-1 1-2 2 4 5 6 7 8-2 9-1 9-2 10 11
kDa MW Sipyloidea sipylus
~70
~55
~35
1 2 3-2 4 9
kDa MW R.artemis
~100
~70
~55
12-2 12-3 9-2
kDa MW S.sipylus
~70
~55
6 7 8-1 8-2 8-3

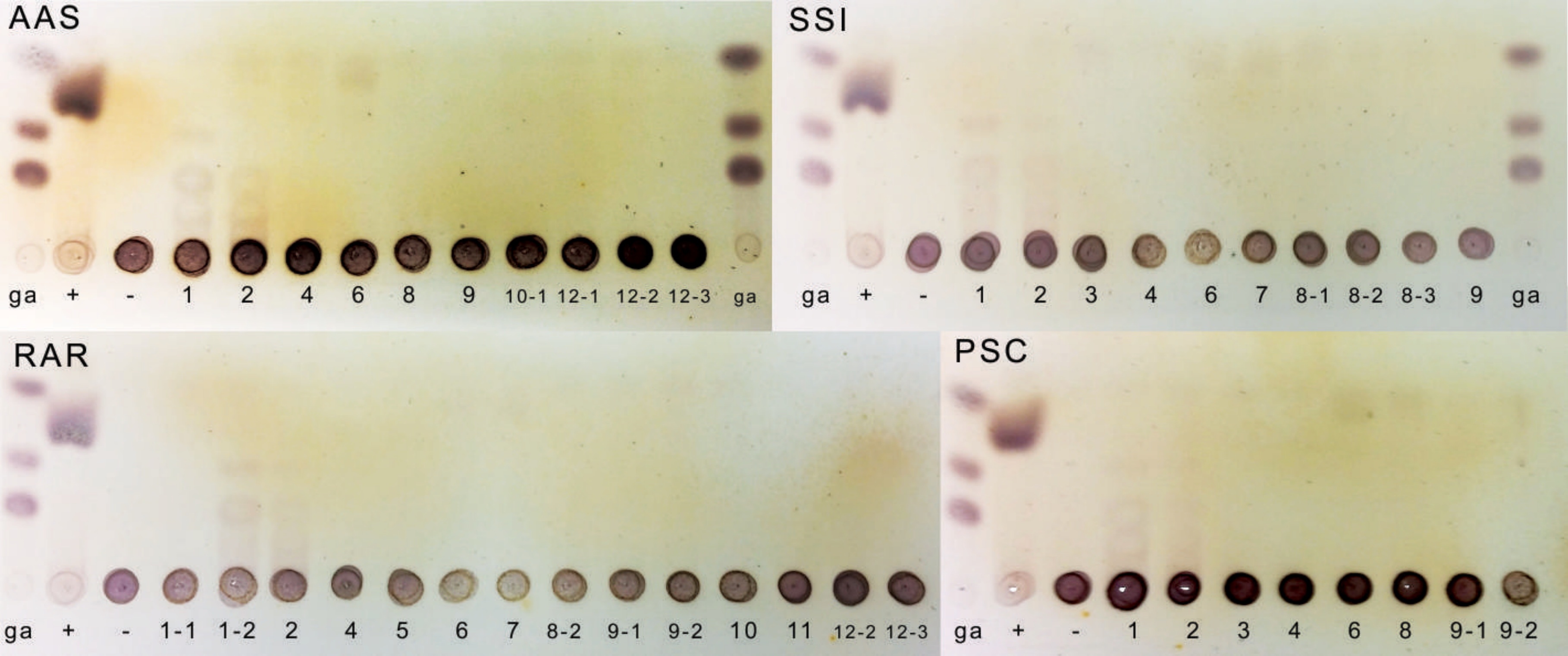
AAS
ga + - 1 2 4 6 8 9 10-1 12-1 12-2 12-3 ga
SSI
ga + - 1 2 3 4 6 7 8-1 8-2 8-3 9 ga
RAR
ga + - 1-1 1-2 2 4 5 6 7 8-2 9-1 9-2 10 11 12-2 12-3
PSC
ga + - 1 2 3 4 6 8 9-1 9-2

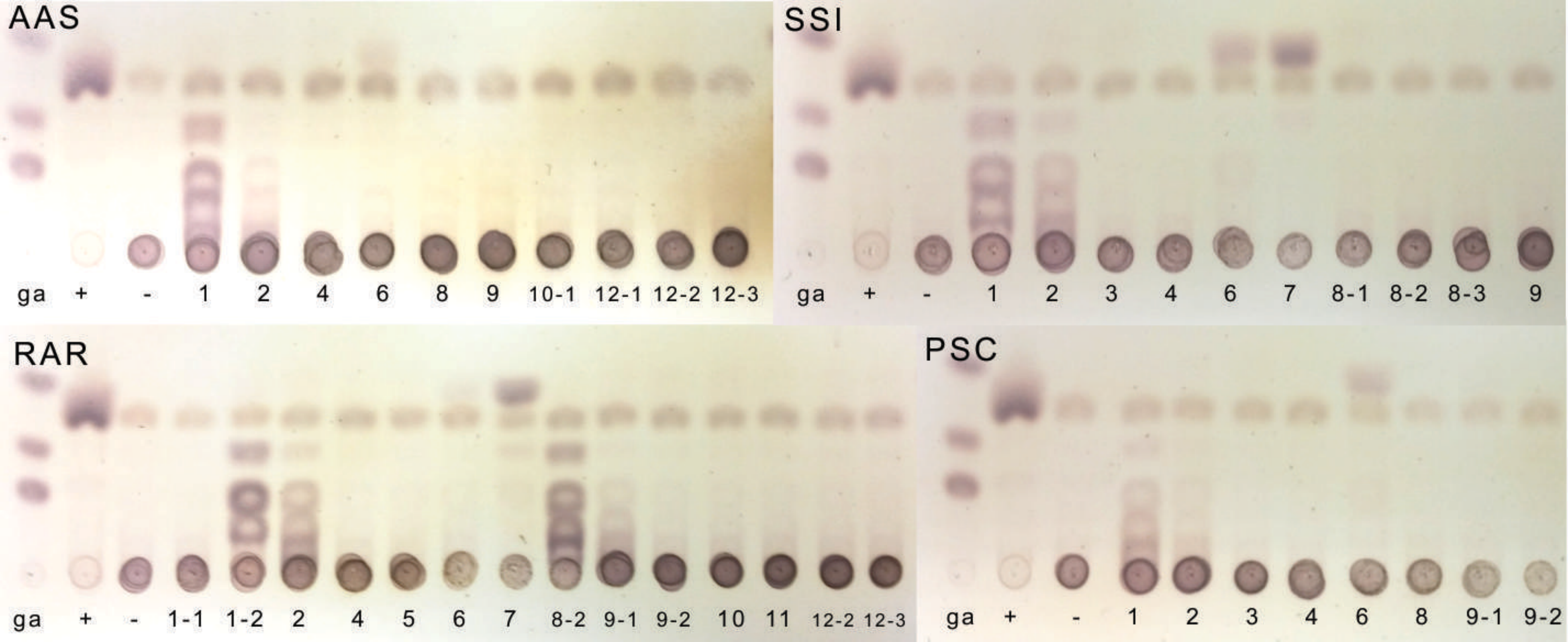

AAS
ga + - 1 2 4 6 8 9 10-1 12-1 12-2 12-3
SSI
ga + - 1 2 3 4 6 7 8-1 8-2 8-3 9
RAR
ga + - 1-1 1-2 2 4 5 6 7 8-2 9-1 9-2 10 11 12-2 12-3
PSC
ga + - 1 2 3 4 6 8 9-1 9-2

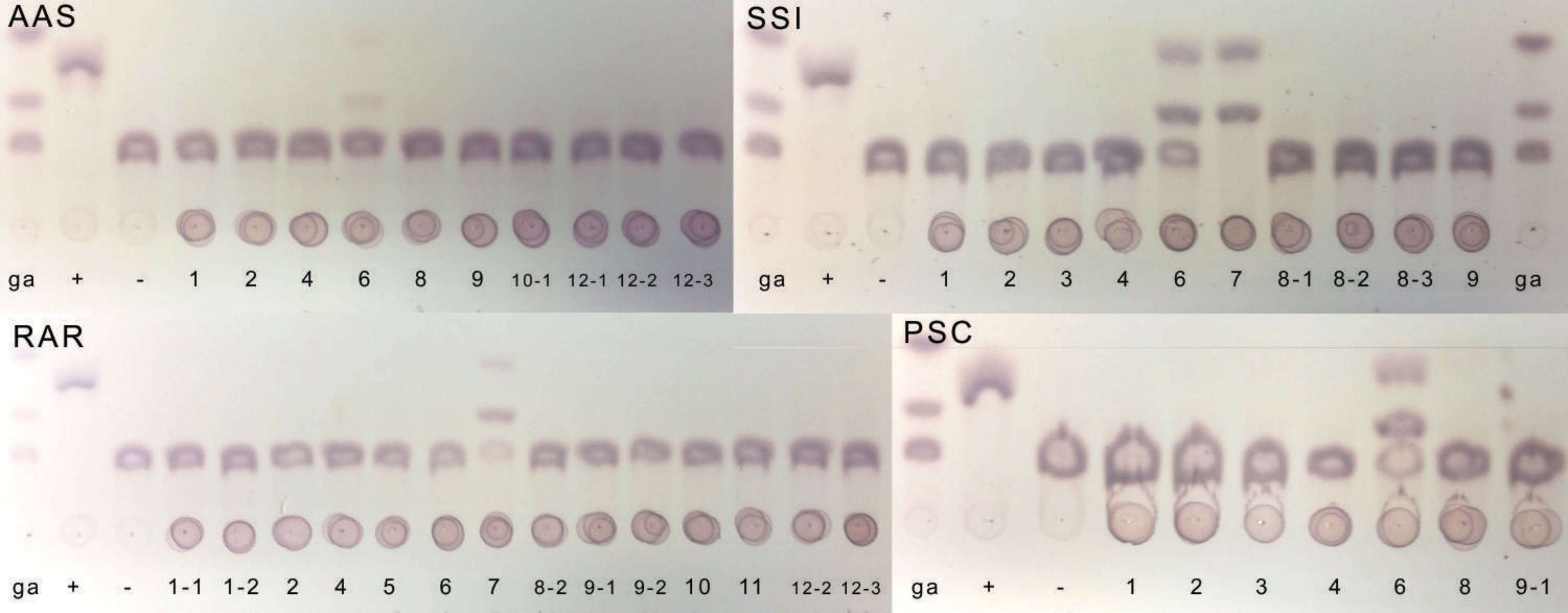
AAS
ga + - 1 2 4 6 8 9 10-1 12-1 12-2 12-3
SSI
ga + - 1 2 3 4 6 7 8-1 8-2 8-3 9 ga
RAR
ga + - 1-1 1-2 2 4 5 6 7 8-2 9-1 9-2 10 11 12-2 12-3
PSC
ga + - 1 2 3 4 6 8 9-1

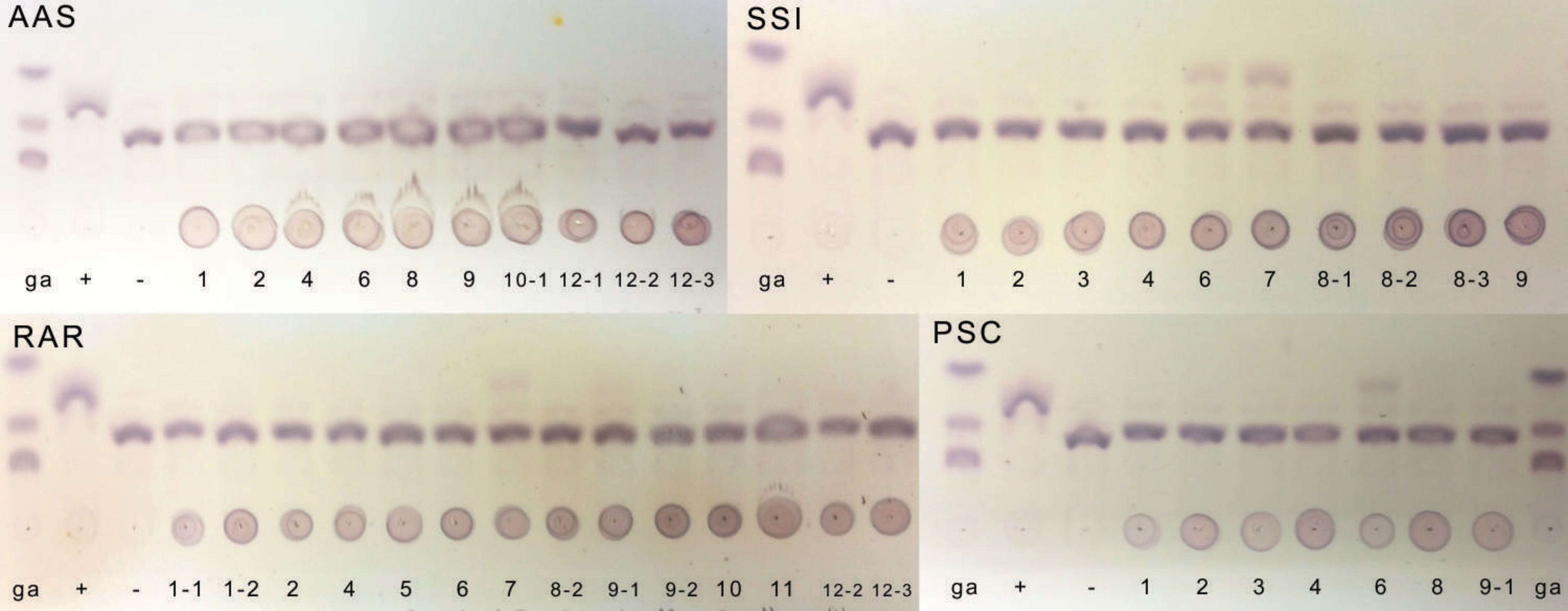

AAS
ga + - 1 2 4 6 8 9 10-1 12-1 12-2 12-3
SSI
ga + - 1 2 3 4 6 7 8-1 8-2 8-3 9
RAR
ga + - 1-1 1-2 2 4 5 6 7 8-2 9-1 9-2 10 11 12-2 12-3
PSC
ga + - 1 2 3 4 6 8 9-1 ga

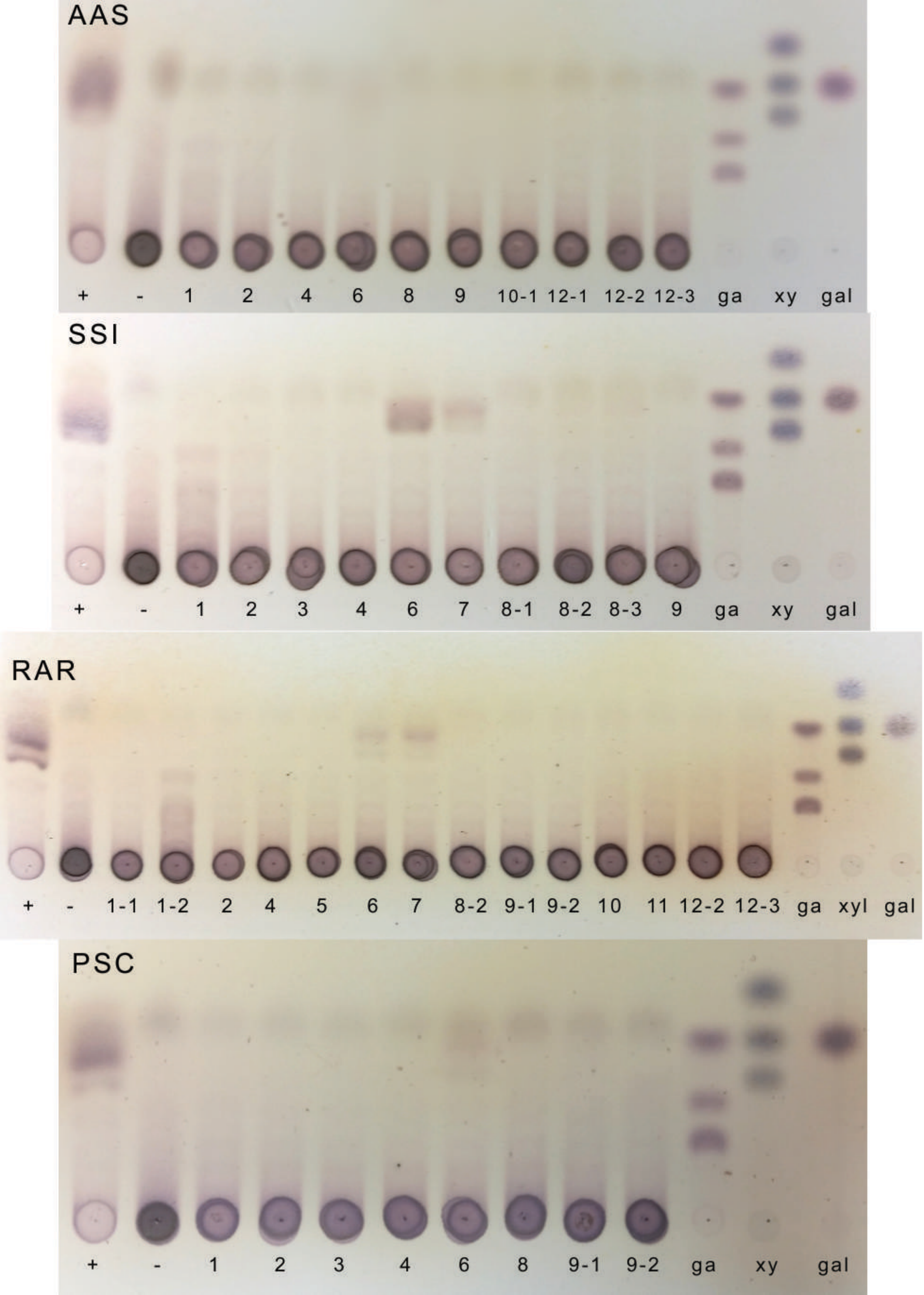
AAS
+ - 1 2 4 6 8 9 10-1 12-1 12-2 12-3 ga xy gal
SSI
+ - 1 2 3 4 6 7 8-1 8-2 8-3 9 ga xy gal
RAR
+ - 1-1 1-2 2 4 5 6 7 8-2 9-1 9-2 10 11 12-2 12-3 ga xyl gal
PSC
+ - 1 2 3 4 6 8 9-1 9-2 ga xy gal

Citrus Pectin

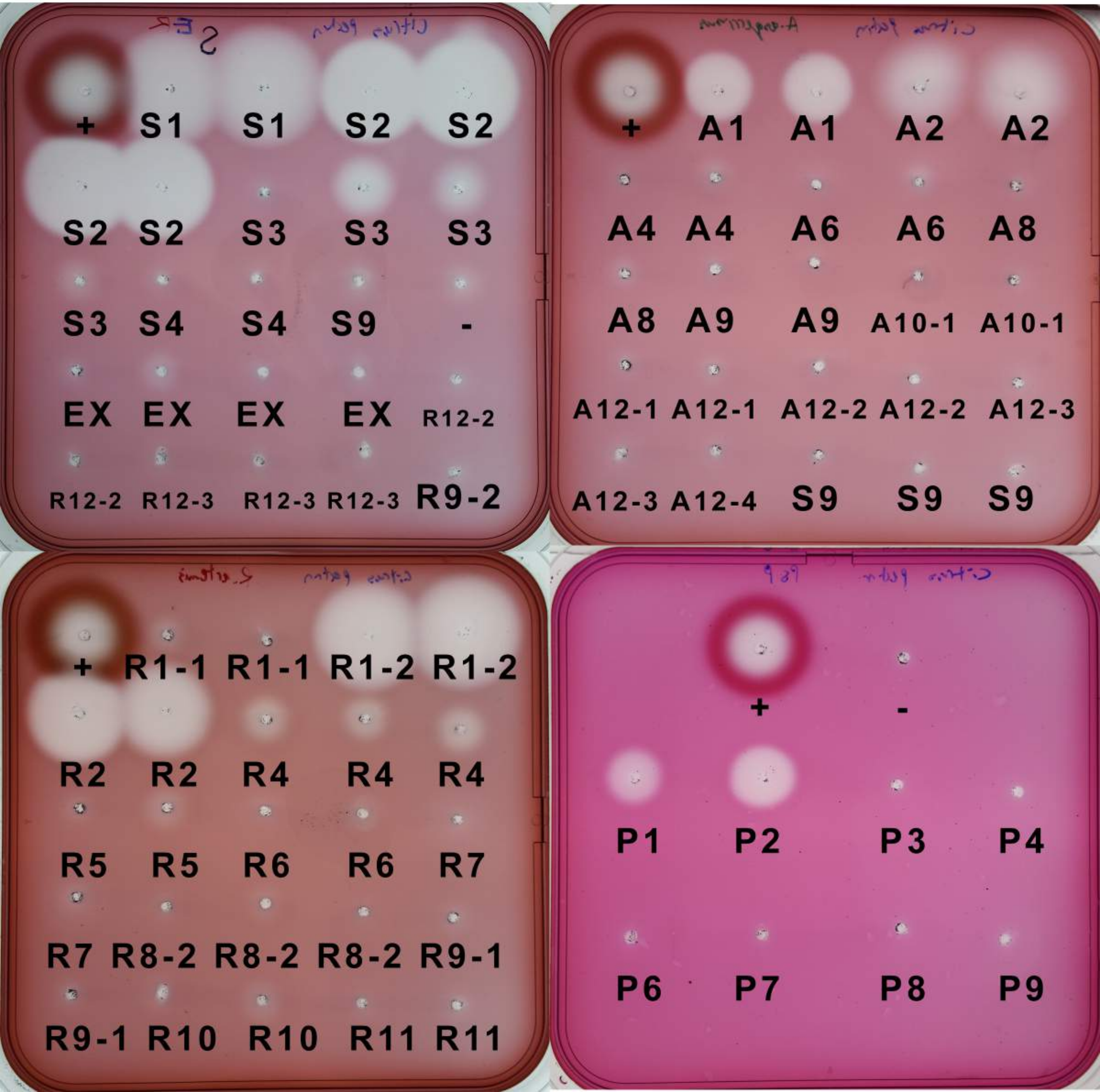

Polygalacturonic Acid

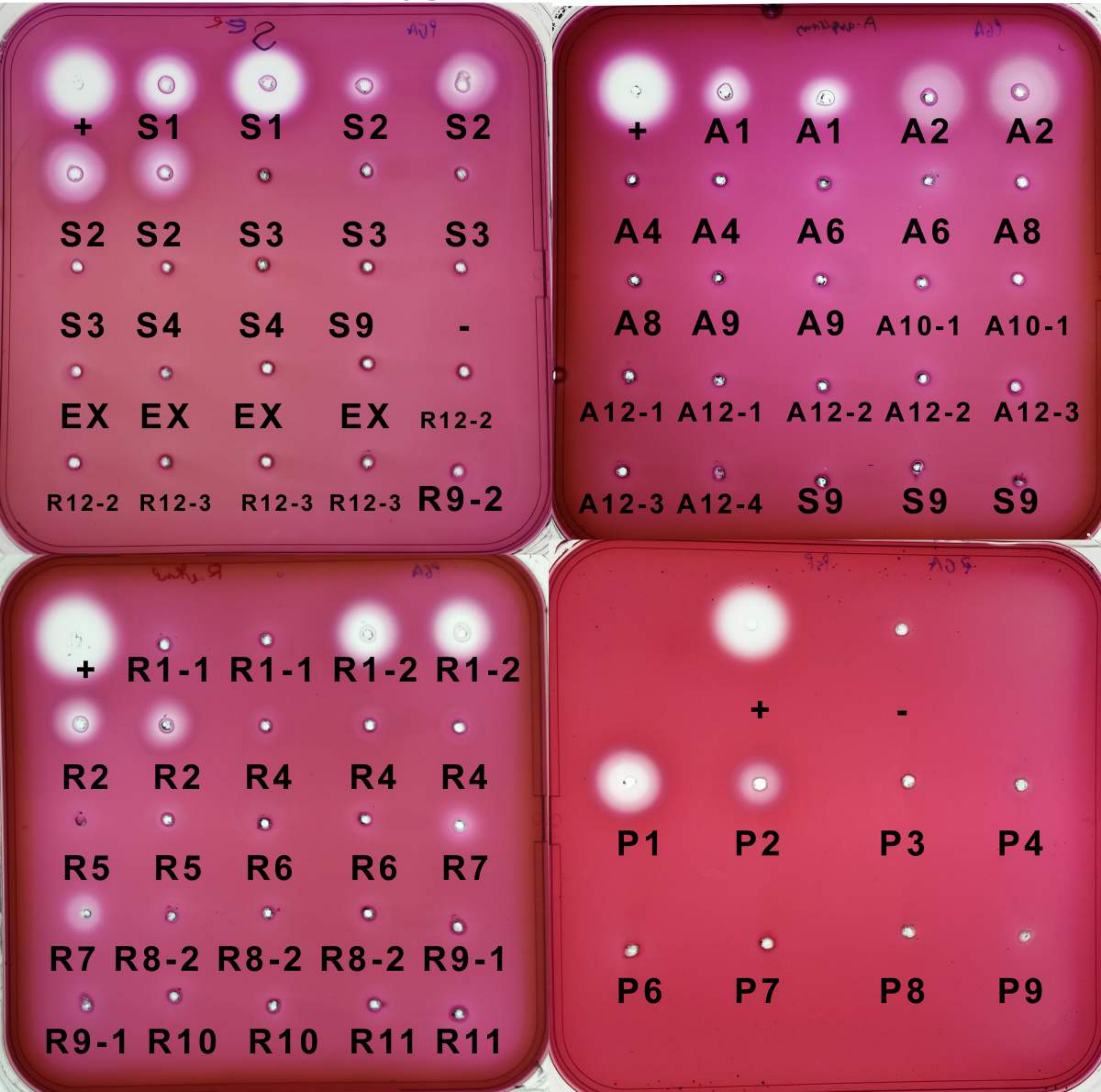

Supplement: Supplementary Information [file srep26388-s1.pdf]
